# Abstract of the Proceedings of the Buffalo Medical Association, March 2, 1875

**Published:** 1875-04

**Authors:** E. N. Brush

**Affiliations:** Secretary pro tem.


ART. II.—*Abstract of the Proceedings of the Buffalo Medical Association, March 2d,* 1875. Reported by E. N. Brush, M. D., Secretary pro tem.

The President, Dr. James P. White, in the chair.

Members present: Drs. White, Rochester, Gould, J. F. Miner, Fowler, W. W. Miner, Wetmore, Potter, Brecht, Briggs, Walsh and Brush.

On motion, Dr. E. N. BRUSH was elected Secretary pro tem.

The minutes of the previous meeting were read and approved.

The applications for membership of Drs. Willoughby and Bielby were taken from the table, and they were elected members on complience with the by-laws. The application of Dr. Lucien Howe was received, and under the rules laid upon the table for one month.

On motion of Dr. FOWLER, Dr. Dorr was invited to participate in the meetings until he could become a member.

Dr. J. F. MINER presented the picture of a little girl who had been sent to him from Darien for an operation. The child was four years of age, and had a tumor situated at the lower end of the spinal column, which was at first thought to be spina bifida, but finally the conclusion was arrived at that it was fibro-cystic in character, and its removal decided upon. The operation was made at the Sisters Hospital, February 17th, and he presented for inspection the bones of a human lower extremity which were taken from the tumor after its removal.*

Dr. ROCHESTER thought that Dr. Miner had not done himself justice in his report of the case. He gave the patient a very thorough and careful examination, and throughout the operation was very careful as to where he cut, especially was this noticable when the rectum was exposed, Dr. Miner being very careful in dissecting the tumor from its walls.

Dr. WHITE remarked that the whole subject of embryology was full of interest. He remarked that monstrosities were of two kinds, by exclusion and inclusion. Each ovum has its separate sac, but it occasionally occurs that they are included in one. In the developement of the fœtus as the anterior or posterior arches become fully developed, and unite one or the other occasionally includes the whole or a portion of another fœtus which may be within the uterine cavity; or two germs may become welded, as it were, together as was illustrated by a monstrosity in Italy. It had two heads and the upper extremeties to correspond, but terminated in two feet only, to these children were given the names of Rita and Christina, and they seemed to have an independent exist-

*See page 321 of this number of Journal, Clinical Remarks, Case XVII.

ence, for instance one would sleep while the other was awake, or one cry while the other laughed. If one foot was tickled the corresponding child would laugh while the other would not appear to notice it. Inclusion is sometimes partial, at others entire. A case is recorded in the *Gentlemans Magazine* of 1813 or 1814, in which a boy had a cyst depending from his perineum nearly to his toes, which contained the remains of another fœtus. Velpeau mentions a case in which there were depending from the abdomen of a boy the lower portion of a fœtus from the unbilical region down. Others are related where the head protruded. Ambrose Pare and others mention cases of this kind. Pare also mentions a man aged forty, who exhibited himself in Paris, who had hanging from his abdomen a fœtus perfectly developed from the shoulders down. A Chineese boy, A-Ki, was once exhibited in France, having a similar condition of things. Several of these cases are well authenticated, others are to be received with some allowance.

The remarkable feature of this case was that while the foot and leg was well developed, the pelvic bone, which was the only portion present of the opposite side, was very deficient in size.

Dr. MINER made a few remarks concerning tumors containing what were apparently fœtal remains, and spoke of the distinctions which should be made between tumors of this kind and Dermoid Cysts, such as were sometimes found in the ovary, testicle and other localities. He mentioned in brief a case of Dermoid Cyst of the ovary which he had removed.*

He remarked that he wished to present a brief account of an operation which he had performed at the Sisters Hospital on Tuesday the 23d of February.† The patient, a boy, twelve years old, had suffered from infancy with urinary difficulties, and presented many of the symptoms of calculus. Careful and repeated examination at last revealed the presence of stone, and an operation was decided upon. The method chosen in this instance was one which he had previously used on young patients, namely the median or what is now known as Allarton's operation, from an English surgeon, who had brought it into more general notice. Gouley, Little and Markoe had brought it into notice in this country.

*Buffalo Med. and Surg. Journal, Vol. XII, No. 5, p. 164, Ovariotomy by Enucleation.
†See page 324 of this number of Journal, Clinical Remarks, Case XVIII.

He spoke of it as being an illustration of the enormous extent to which the neck of the bladder and urethra is capable of dilatation. The stone which weighed 706 grains and measured 2⅛ in. in length, 1½ in. in breadth, and 1⅛ in. in thickness, was removed in fifteen minutes, which was perhaps a little too rapid, although no accident happened, it is better to make the dilatation a little slower. As the stone lay in the forceps it measured 1⅝ inches in one diameter (over the forceps) by 1⅛ inches between the blades.

Dr. WETWORE asked if Dolbeau had not first recommended the vertical incision.

Dr. MINER replied that Dolbeau had made some modification of the operation, one of which was the employment of a branched dilator by which the limit of dilation is fixed.

Dr. WHITE spoke of the extent to which the female urethra could be dilated. He had removed in this way a stone from the female bladder larger than the one exhibited, and others, but of a smaller size. He did not doubt but that this operation was possible in young males, but thought that in adult life when the prostate was enlarged and indurated it would be impossible.

Dr. MINER replied that in England it had been abandoned in the adult, lithotrity or the lateral operation being preferable.

Dr. ROCHESTER said that he had seen many operations for stone, and was present at this one. He wished to bear testimony to the ease and facility with which it was made. It was somewhat of a surprise to him that so large a stone could be extracted with no more cutting than was here employed.

PREVAILING DISEASES.—The general opinion seemed to be that there was less scarlet fever than there had been. Some erysipelas was reported. After a short discussion upon the contagious properties of certain diseases, the meeting was declared adjourned.

